# Decaprenyl-phosphoryl-ribose 2′-epimerase (DprE1): challenging target for antitubercular drug discovery

**DOI:** 10.1186/s13065-018-0441-2

**Published:** 2018-06-23

**Authors:** Jineetkumar Gawad, Chandrakant Bonde

**Affiliations:** Department of Pharmaceutical Chemistry, SVKM’s NMIMS School of Pharmacy & Technology Management, Shirpur Dist, Dhule, Maharashtra 425 405 India

**Keywords:** DprE1, Antitubercular agents, Covalent and non covalent inhibitors, Future needs

## Abstract

Tuberculosis has proved harmful to the entire history of mankind from past several decades. Decaprenyl-phosphoryl-ribose 2′-epimerase (DprE1) is a recent target which was identified in 2009 but unfortunately it is neither explored nor crossed phase II. In past several decades few targets were identified for effective antitubercular drug discovery. Resistance is the major problem for effective antitubercular drug discovery. Arabinose is constituent of mycobacterium cell wall. Biosynthesis of arabinose is FAD dependant two step epimerisation reaction which is catalysed by DprE1 and DprE2 flavoprotein enzymes. The current review is mainly emphases on DprE1 as a perspective challenge for further research.

## Introduction

Tuberculosis (TB) is a major worldwide concern whose control has become more critical due to HIV and increased multidrug-resistance (MDR-TB) and extensively drug resistance (XDR-TB) strains of *Mycobacterium tuberculosis* [1]. The need for newer and effective antiTB drugs are more essential. In the previous decade hard efforts have been made to find new leads for TB drug development utilizing both target-based and structure-based methodologies [2]. Here, we have emphasized on few covalent and non-covalent Decaprenyl-phosphoryl-ribose 2′-epimerase (DprE1) inhibitors which might play the important role in most useful antitubercular therapies those are in clinical advancement. DprE1, an enzyme protein associated with a vital step of epimerisation in mycobacterial cell wall biosynthesis [1].

*Mycobacterium tuberculosis* is one of the world’s most dreadful human pathogen because of its ability to persist inside humans for longer time period in a clinically inactive state. Roughly 95% of the general population who infected (33% of the worldwide population) built up an inert infection. The current available vaccine, *Mycobacterium bovis* Bacillus Calmette–Guerin (BCG), is mostly used in recent years. Specifically, this vaccine prevent most serious types of the infection and not from disease. *M. tuberculosis* stimulates a solid response, however it has advanced to oppose the body’s activities to kill it and regardless of the possibility of underlying disease is effectively controlled, many people built up an inactive infection that can hold on for quite a long time [3]. For example, Aagaard and colleagues [4] have built up a multistage immunization technique in which the early antigens Ag85B and 6-kDa early secretory antigenic target are joined with the inertness related protein Rv2660c (H56 antibody). In two mouse models of dormant tuberculosis, they demonstrated that, H56 immunization after presentation can control reactivation and altogether bring down the bacterial load contrasted with adjuvant control mice. The discovery of drugs with novel mechanism of action is direly required because of the expanding number of multidrug safe (MDR), which are strains of *M. tuberculosis* that are resistant to both isoniazid and rifampicin, with or without protection from different medications, broadly XDR and MDR strains additionally resistant to any fluoroquinolone and any of the second-line against TB injectable medications (amikacin, kanamycin, or capreomycin) [5].

Mycobacteria are resistant to regular antibiotics with the few exceptions of aminoglycosides, rifamycins and fluoroquinolones [6]. General resistance from therapeutic agents is identified with the structure of the mycobacterial cell envelope bringing about low permeability to exogenous factors [7]. Therefore, a few chemotherapeutic operators are active against Mtb were created. After streptomycin—the primary antitubercular agent and 4-aminosalicylic acid in the 1940s, isoniazid was presented in 1952 and still is the significant component of the antibiotic treatment of TB, WHO groups first-line and second-line antitubercular operators relying on their adequacy and resistance [8].

## Decaprenyl-phosphoribose 2′-epimerase (DprE1)

The heteromeric protein decaprenyl-phospho-ribose 2′-epimerase catalyzes the epimerization reaction of decaprenylphosphoryl-d-ribose (DPR) into decaprenylphosphoryl-d-arabinose (DPA) [9]. This reaction occurs through a successive oxidation–reduction involving the intermediate (decaprenylphosphoryl-2-keto-β-d-erythro-pentofuranose, DPX), which is a result of DPR oxidation and a precursor of DPA [10]. This compound is made up of two proteins encoded by the DprE1 and DprE2 genes. DprE1 and DprE2 have been recommended as decaprenylphosphoryl-β-d-ribose oxidase and decaprenylphosphoryl-d-2-keto erythro pentose reductase, separation [11]. Trefzer and collaborators announced the in vitro interpretation of the enzymatic exercises of sanitized recombinant DprE1 and DprE2 orthologous proteins from *Mycobacterium smegmatis* and exhibited that DprE1 goes about as an oxidase and DprE2 as a reductase [12]. For epimerase activity, a synchronous articulation of the two polypeptides is required [13].

## Crystal structure of DprE1

Three structures of *Mycobacterium smegmatis* DprE1 have been established in two distinctive groups and one structure contains BTZ043 [14]. The 19 different structures are *M. tuberculosis* DprE1 solidified, to be specific hexagonal and orthorhombic, in complex with or without inhibitors [15]. DprE1 is represented by the two-domain topology of the vanillyl-liquor oxidase group of oxido-reductases including a FAD-restricting area and the substrate-restricting ares [16]. The monoclinic and hexagonal precious stone structures show an obvious dimer of DprE1. In any case [14], DprE1 does not dimerise in solution. The cofactor is profoundly covered in the FAD-restricting area, with the isoalloxazine present at the interface of the substrate-restricting space before the substrate-restricting pocket [17]. As contrast to the homologous structure of alditol oxidase, DprE1 does not covalently tie the prosthetic assembly. Intriguingly, the *M. smegmatis* DprE1 structure has likewise been understood without the FAD cofactor, showing that FAD is inessential for the collapsing of the protein. Electron density in all crystal structures acquired for *M. tuberculosis* or *M. smegmatis* [18].

## Inhibitors of DprE1

BTZ043, the lead compound of the benzothiazinone (BTZ) series, was the primary DprE1 inhibitor described and is particularly strong with an in vitro or in vivo minimum inhibitory concentration (MIC) in the nanomolar extend [19]. The mechanism of BTZ043 clarifies its significant strength since it carries on as a suicide substrate for the decreased type of DprE1 [20]. BTZ043 and other BTZ series experience nitroreduction to nitroso derivatives, which particularly frames a covalent adduct with cysteine 387 (C387) in the dynamic site of DprE1, irreversibly hindering the protein [21]. C387 is profoundly saved in orthologous DprE1 chemicals in actinobacteria, aside from in *Mycobacterium avium* and *Mycobacterium aurum* where cysteine is supplanted by alanine and serine individually. These transformations present characteristic BTZ protection [22].

## Current status of DprE1 inhibitors

To date, 15 new classes of DprE1 inhibitors with antimycobacterial activity have been reported. These inhibitors are categories into two families as per their method of activity (Table 1). Six are known to inhibit DprE1 irreversibly by framing a covalent adduct with C387 of DprE1 in an indistinguishable way from BTZ, though nine as competitive non-covalent inhibitors [23]. Regular characteristics of the covalent inhibitors are the close of a nitro group and their potency against C387A and C387S DprE1 mutants [24]. PBTZ169 has one of the most minimal MICs against *M. tuberculosis* (0.6 nM) and came out because of a lead optimisation process. PBTZ169 has finished phase I clinical trials and acts in cooperative energy with pyrazinamide and bedaquiline [25]. DprE1 has similarly been distinguished as the objective of the dinitrobenzamides (DNBs), nitroquinoxalines (with the lead atom VI-9376) and nitroimidazoles (with the lead particle 377790), all of which act as covalent inhibitors. As of late, another framework was found from an entire cell screen against *Mycobacterium bovis* BCG which brought about the identification of benzothiazole-*N*-oxide (BTO) focusing on DprE1 [26]. Unfortunately, a few toxicity qualities and mutagenicity issues were related with this molecule. Be that as it may, regulating the stereoelectronic properties of the benzothiazole ring in SAR thinks about prompted the revelation of a novel class of antitubercular operators called cBT [6-methyl-7-nitro-5-(trifluoromethyl)-1,3-benzothiazoles]. Although less potent, cBT are non-mutagenic and show an enhanced safety characteristics [27]. Genotoxicity is a major concern for covalent inhibitors on the grounds that nitro aromatic compounds by and large convey a danger of mutagenicity; PBTZ169 has been observed to be non-mutagenic in preclinical tests [28]. Two investigations have exhibited that the nitro group show on BTZ can be supplanted with a pyrrole ring or an azide group while at the same time holding critical antimycobacterial action. These non-nitro BTZ analogues at that point carry on as non-covalent inhibitors and are significantly less strong than their covalent counterparts [29]. Within the previous 3 years, an impressive number of non-covalent DprE1 inhibitors have been found. In a cell-based screen, another compound, TCA1, was recognized that has action against replicating and no replicating *M. tuberculosis*. It is likewise powerful in vivo alone or in combination with bleeding edge TB medicates in acute and chronic mouse models of TB [30]. Without a nitro group, it can’t tie covalently to C387. TCA1-resistant mutants harbour Y314C substitution in DprE1. Interestingly, the Y314C mutant strain, which is resistant to TCA1, stays selective to BTZ recommending that the coupling component of TCA1 to DprE1 is not the same as that of BTZ. As of late, new molecules in light of the structure of TCA1, benzothiazolylpyrimidine-5-carboxamides, were outlined by structure-based drug design approaches [31]. These new molecules are more dynamic in action than TCA1 with a MIC of 80 nM (seven-overlap lower than that of TCA1) in *M. tuberculosis* and have preferred oral bioavailability over TCA1 and BTZ043. The 1,4-azaindole arrangement is another class of non-covalent inhibitors that target DprE1, and was distinguished among a framework transforming approach beginning from a distributed against TB, non-DprE1 imidazo-pyridine scaffold. Spontaneous resistance mutants contain a single Y314H change in DprE1, no cross resistance was seen amongst BTZ and azaindole-resistance strains, recommending that TCA1 and 1,4-azaindoles carry on also to non-covalent inhibitors [32]. Pyrazolopyridones, which began from an entire cell screen, were likewise found to restrain DprE1 in a non-covalently with a MIC of 0.1 mM. Similarly as with 1,4-azaindoles, the Y314H transformation gives protection from pyrazolopyridones. Interestingly, pyrazolopyridones demonstrated improved strength against the BTZ-resistant strains conveying C387S and C387G changes in DprE1 as compared with the wild type strain. This arrangement has not been tried in vivo in light of the fact that the pharmacodynamic properties required for further optimization [33].

**Table 1:** Covalent and non-covalent DprE1inhibitors

Covalent inhibitors			Non covalent inhibitors		
Compound	Structure	References	Compound	Structure	References
BTZ043		[23]	1–4 Azaindoles		[34]
DNB1		[35]	2-Carboxyquinoxalines		[32]
377790		[26]	4-AQs		[36]
cBT		[16]	8-Pyrrole-BTZ		[37]
BTO		[38]	1,3-BTZ azide		[39]
VI-9376		[40]	Pyrazolopyridones		[33]
PBTZ		[17]	1,2,4-Triazole containing 1,4-BTZ derivatives		[41]

## Structural studies of DprE1 in complex with covalent inhibitors

*Mycobacterium smegmatis* DprE1 was crystallised in complex with BTZ043, revealing insight into the basic principle of the inhibition mechanisms of covalent inhibitors [14]. BTZ043 is a component based covalent inhibitor, which requires the enzymatic action of the protein with the substrate to change over the nitro group of BTZ043 to get the structure of the covalent complex, DprE1 was incubated with BTZ043 and farnesylphosphoryl-d-ribofuranose (FPR; a simple of DPR with a shorter polyprenyl chain filling in as a reasonable chemical substrate) before performing crystallisation trials [38]. BTZ043 is situated in the substrate-restricting pocket before the isoalloxazine ring of FAD and ties covalently to C394 (proportional to C387 in *M. tuberculosis*). There are no major basic changes between the local complex types of DprE1 [17]. The trifluoromethyl group of BTZ043 is arranged in a hydrophobic pocket framed by side chains of H132, G133, K134, K367, F369 and N385. The piperidine ring of BTZ043 is kept up on each side by the isoalloxazine ring of FAD, and by G117 and V365. The spirocyclic moiety of BTZ043 is situated at the protein surface and needs full electron thickness, bringing about the adaptability of this area of the particle. To be sure, there is just a single van der Waals interaction amongst L363 and the spirocyclic moiety [32].

## Benzothiazinones

**Fig. 1:**
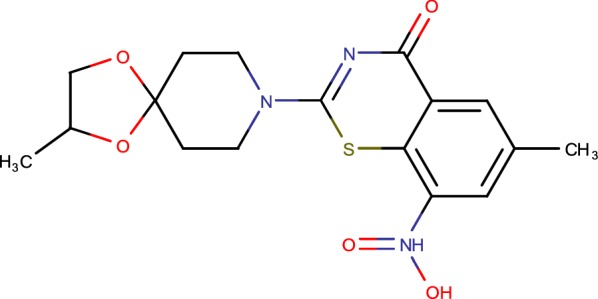
Benzothiazinones

The main class of derivatives focusing on DprE1 is that of BTZs (Fig. 1), a development of sulfur-containing heterocycle mixed with antibacterial action. The MIC range of synthesized BTZs against various mycobacteria ranges from ~ 0.1 to 80 ng/ml for quick producers and from 1 to 30 ng/ml for individuals from the *M. tuberculosis* complex. The MICs of BTZ043 against *M. tuberculosis* H37Rv and *M. smegmatis* were 1 and 4 ng/ml, respectively [23]. BTZ043 is bactericidal, diminishing feasibility in vitro by more than 1000 folds in less than 72 h. The take-up, intracellular killing, and potential cytotoxicity of BTZ mixes in an in vivo model were resolved. Macrophages regarded with BTZ043 were ensured by comparing and those treated with the negative controls [38].

In the greater part of the drug resistance mutants inspected, a similar codon of dprE1 was influenced: the cysteine at position 387 was supplanted by serine or glycine codons, separately. The BTZ protection deciding district of dprE1 was profoundly saved in orthologous qualities from different Actinobacteria, aside from that in a couple of situations where Cys387 was replaced by serine or alanine. The comparing microscopic organisms, *Mycobacterium avium* and *Mycobacterium aurum*, were observed to be normally resistant to BTZ, in this way supporting the distinguishing proof of DprE1 as the BTZ target [23]. As early metabolic investigations with microscopic organisms or mice demonstrated that the BTZ nitro group could be lessened to an amino group, the S and R enantiomers of the amino groups and the imaginable hydroxylamine middle were incorporated and tried for antimycobacterial action. The amino and hydroxylamine groups were significantly less dynamic regard to the nitro group. Regarding this, a protection mechanism to BTZs was represented in *M. smegmatis* [42]. The overexpression of the nitroreductase NfnB prompts the inactivation of the medication by lessening of a basic nitro group to an amino group. Some *M. smegmatis* BTZ-safe mutants which harbored neither changes in MSMEG_6382 (dprE1) nor in MSMEG_6385 (dprE2), however in the MSMEG_6503 quality, coding for a putative transcriptional controller from the TetR family were separated. It unveiled that this controller controls the translation of the MSMEG_6505 quality, coding for NfnB compound. This transformation prompted a flawed repressor, causing overexpression of NfnB and thus, the decrease of the BTZ nitro atom to its less dynamic amino group [42]. To additionally the immediate part of NfnB in the BTZ protection, an in-outline unmarked cancellation was made in the nfnB quality and the ΔnfnB strain was touchy to BTZ [10].

## Dinitrobenzamides

**Fig. 2:**
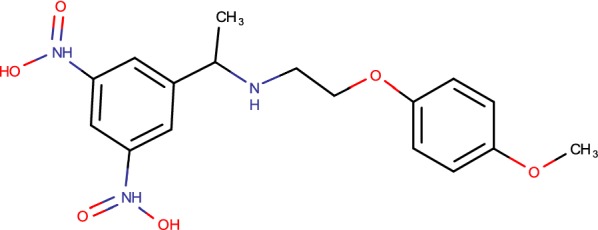
Dinitrobenzamides

A couple of months after the publication of BTZs as promising antitubercular drug focusing on DprE1 another new class, the DNB derivative (Fig. 2) were distinguished through a screening of chemicals which interfere with *M. tuberculosis* replication inside macrophages [35]. The method developed depends on the utilization of automated confocal fluorescent microscopy to screen intracellular development of green fluorescent protein-expressing *M. tuberculosis* H37Rv in Raw264.7 macrophages. The screening of a library of more than 50,000 small compounds led to the identification of 135 active and non-toxic compounds. These compounds had a MIC of around 70 ng/ml, which is like that of isoniazid [35]. To recognize the chemical substituents important for benzamide antibacterial action, more than 155 extra compounds were synthesized and their structure–activity relationship was broke down utilizing both intracellular and in vitro development assays. The two notable compounds from this series [*N*-(2-(4-methoxyphenoxy)ethyl)-3,5-dinitrobenzamide] and [*N*-(2-(benzyloxy)ethyl)-3,5-dinitrobenzamide] named DNB1 and DNB2, separately, were sought after further since their exercises on intracellular and extracellular *M. tuberculosis* were especially ideal. No cell poisonous quality was noted for these mixes utilizing customary cytotoxicity tests of uninfected cells [21]. Investigation of the wide antimicrobial range uncovered that the impact of these DNB derivatives were principally confined to Actinomycetes, with the potent activity observed against *Mycobacterium* with a MIC of 75 ng/ml. DNB1 and DNB2 were additionally very active against MDR and XDR TB clinical isolates. Besides, these two compounds were additionally connected with low levels of unconstrained protection. The bactericidal impact on *M. tuberculosis* of DNB1 and DNB2 was observed to be time active and to require a few days to achieve bacterial clearance, inferring that they could interfere with de novo mycobacterial biosynthesis. This was additionally verified by the way that the DNB derivatives lost their action in a non-replicating *M. tuberculosis* framework [21]. To pick up knowledge into the feasible focuses of DNBs, the impact of DNB1 and DNB2 on the lipid organization of the cell envelope of *M. tuberculosis* was examined; no impacts on the biosynthesis of unsaturated fats, mycolic acids, as well as different lipids were noted. By difference, DNB1 and DNB2 demonstrated, a clear impact on the blend of the arabinan part of arabinogalactan and lipoproteins. The impacts of DNB in the interference of the combination of DPA were tried. Examinations uncovered the finish hindrance of DPA development in the DNB-treated concentrates, simultaneous with the aggregation of DPR, showing that the objective of DNBs could be the heteromeric decaprenylphospho-ribose epimerase encoded by the dprE1/dprE2 qualities in *M. tuberculosis* H37Rv. Besides, BTZ-safe mutants of *M. smegmatis* and *M. bovis* BCG, having a transformation in dprE1 quality, were additionally impervious to DNBs. This theory has been as of late stated demonstrating that the DNBs and the BTZs have an indistinguishable focus from well as similar systems of protection [43]. Specifically, to better comprehend the system of protection from DNBs, a few unconstrained *M. smegmatis* mutants impervious to *N*-(2-(3-chlorobenzyloxy)ethyl)-3,5-dinitrobenzamide (DNB3) were secluded. DNB3 was selected due to its higher solubility in acid medium in regard to alternate DNBs derivatives. The unconstrained mutants displayed two diverse protection levels to DNB3. The main arrangement of *M. smegmatis* mutants demonstrated an abnormal state of protection from DNBs and the second arrangement a lower level of protection (64–128-overlap the MIC). The possible cross resistance amongst DNBs, BTZs was checked and exhibited for all *M. smegmatis* mutants [44].

## PBTZ169

**Fig. 3:**
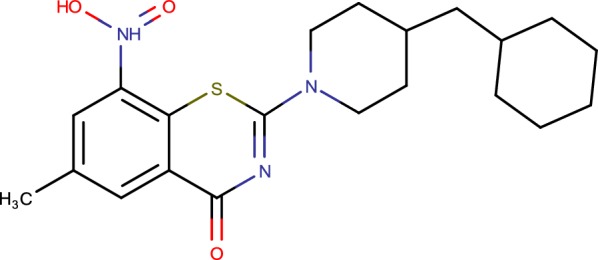
PBTZ169

PBTZ169 is a piperazinobenzothiazinone derivative (Fig. 3) upgraded by therapeutic science from the lead BTZ043. PBTZ169 has a few focal points compared with BTZ043, among which are simpler synthetic blend, because of the absence of chiral centres, cost of goods and better pharmacodynamics [17]. PBTZ169 covalently represses DprE1, a catalyst basic for the biosynthesis of key cell wall components. PBTZ169 has added substance impacts with numerous TB helpful specialists, both promoted and being developed, and has synergic impacts with bedaquiline in preclinical models. The innovative medicines for tuberculosis (iM4TB) establishment is driving PBTZ169 improvement in the Rest of the World [45]. Innovative medicines for tuberculosis (iM4TB) additionally design a phase I has began in Switzerland in 2017. In April 2017, The Bill and Melinda Gates foundation granted EPFL-based non-benefit iM4TB $2.45 million to take their fertile aggressive to tuberculosis tranquilize PBTZ169 into clinical trials [46].

## VI-9376

**Fig. 4:**
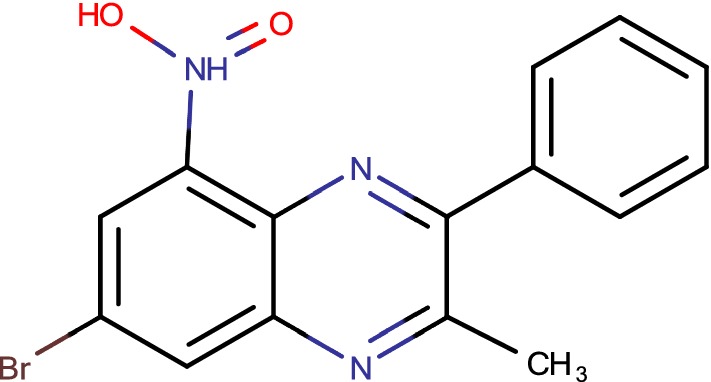
VI-9376

The last class of compounds known to emphasis on the *M. tuberculosis* DprE1 is the benzoquinoxalines (Fig. 4). For this condition, to discover antimycobacterial frameworks, a kinase inhibitor library of more than 12,000 compounds from Vichem Chemie Ltd. was screened using a coordinated system including whole cell-based assays and target based assays with the protein kinase PknA [47]. Actually, signaling pathways in Prokaryotes are additionally controlled by protein kinases. Also, a couple of cases of compounds got from protein kinase pharmacophores have been appeared to repress non-kinase antibacterial targets, for example, d-alanine–d-alanine ligase or biotin carboxylase kinase [48]. In this manner, kinase inhibitor libraries can possibly be a wellspring of inhibitors for an extensive variety of bacterial proteins. Of the 12,100 compounds tested, more than 200 shown promising activity against *C. glutamicum* of which 17 additionally showed activity against *M. tuberculosis*. These 17 compounds were tried for inhibition of PknA and PknB. None of them particularly inhibited the serine/threonine protein kinases action. They were additionally tested for their potential genotoxic and cytotoxic properties, and their MIC against *M. tuberculosis* H37Rv was resolved. Among these, only three compounds were observed to be non-mutagenic, noncytotoxic, and shown a MIC < 10 mM against *M. tuberculosis* H37Rv [8]. Among the compounds active on *M. tuberculosis*, the structure of VI-9376 incompletely looked like that of the BTZs and DNBs. Consequently, VI-9376 was tested against a few BTZ-resistant mutants of mycobacteria and *C. glutamicum*. The MIC comes about uncovered cross-resistance between the BTZ lead compound, BTZ043, and VI-9376. Forty derivatives of VI-9376 in this manner were incorporated and tested against *M. tuberculosis* H37Rv. The structure–activity relationship and MIC information got for the derivatives demonstrated that the nitro group at the fifth position of the quinoxaline framework is totally required for activity. Substitution of the bromine at the fourth position by a trifluoromethyl expanded the strength of the platform, while adjustments at position 2 or position 3 did not prompt a remarkable change of movement against *M. tuberculosis* [49].

## 377790

**Fig. 5:**
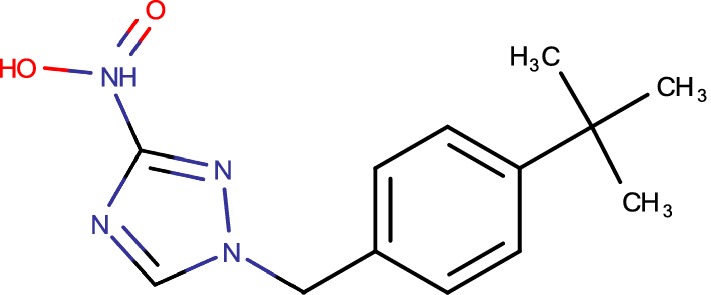
377790

Compound 377790 (1-(4-(tert-butyl)benzyl)-3-nitro-1*H*-1,2,4-triazole) (Fig. 5), a novel nitro-substituted triazole, has great activity against *M. tuberculosis* with an IC90 of 0.5 μM. A few classes of antibiotics that are dynamic against *M. tuberculosis* are described by nitro-substituted heterocycles, including the nitroimidazole metronidazole, different nitrofurans, and the promising new operator PA-824, a bicyclic nitroimidazole [6]. SAR investigation directed around 377790 recommends that the nitro-group is basic for activity, as analogues without the nitro-group, or with different substitutions set up of the nitro bunch are essentially less dynamic than the first nitro-containing hit. The necessity for the nitro group could show that the activity of these compounds requires diminishment of the nitro group to a responsive animal categories, like PA-824, or the nitro group is required for official of the inhibitors to its objective [50]. To explain the component of activity of these derivatives, these derivatives, however fundamentally unlike the triazole derivatives, additionally have a nitro group usefulness, and are thought to restrain DprE1 by development of a covalent security by means of decrease of the nitro group to a nitroso-derivatives that responds particularly with Cys387 [51]. Transformation of Cys387 additionally presents a high level of protection from BTZs. As with BTZs, overexpression of DprE1 presents protection from compounds 377790 and other nitro-triazole analogues additionally confirming that these derivatives likely target DprE1 [26].

## Benzothiazole-*N*-oxide (BTO)

**Fig. 6:**
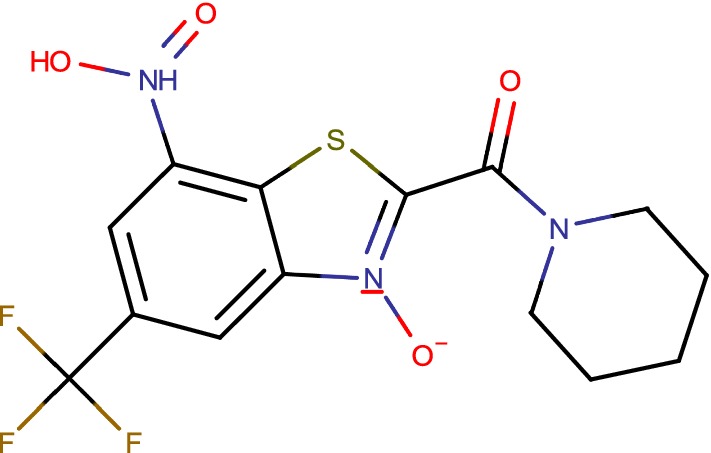
Benzothiazole-*N*-oxide

Report states that non-covalent inhibitors of DprE1 (Fig. 6), were additionally viable in vivo, shown that independent of their method, these inhibitors which annoy the objective inside Mtb possibly assured as future candidate to battle tuberculosis [52]. The screening methodology received here distinguished a novel begin point, the benzothiazoles which displayed strong antimycobacterial properties through particular hindrance of DprE1 [53]. Target linkage was built up by tackling the structure of the benzothiazole—DprE1 complex and its system of hindrance illustrated by showing covalent adduct development with Cys387 at the dynamic site [54]. Quick killing in vitro and in addition proficient intracellular slaughter repeated its antimycobacterial intensity under various development conditions. Information produced against single medication safe strains gave proof of its viability on other clinical detaches [55]. Alongside building a vigorous SAR from BTO to cBT by means of BT that definite the support of strong bactericidal movement through particular hindrance of DprE1, the notable part of this investigation was the utilization of particular therapeutic science approaches for a fruitful change and moderation of mutagenic capability of the first nitro compound to non-genotoxic derivatives. While tending to the mutagenicity, we could make the compounds more secure as for their CYP hindrance potential and could enhance the physicochemical as well as pharmacokinetics properties [44]. We couldn’t test compounds for in vivo adequacy think about as we needed a waitlist of intensifies that would meet a superior lead profile [56]. Also, its pharmacological approval with benzothiazoles as portrayed in this investigation in conjunction with various other reports which have built up its durability offer open doors as potential clinical possibility for improvement against both delicate and medication safe tuberculosis [16].

## 6-Methyl-7-nitro-5-(trifluoromethyl)-1,3-benzothiazoles (cBTs)

**Fig. 7:**
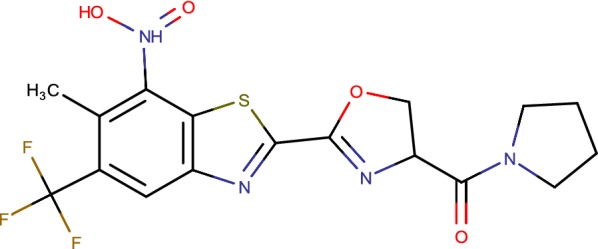
6-Methyl-7-nitro-5-(trifluoromethyl)-1,3-benzothiazoles

Mutagenicity of nitroarenes can be relieved through balance of their stereoelectronic properties. The knowledge into the method of target hindrance and official in conjunction with the electron liking of nitroarenes were observed to be the key components [57]. The distinguishable proof of DprE1 as an objective helped our comprehension of the part of the nitro group. The methyl group that was the key in moderating the mutagenic properties was endured in the dynamic site of the protein. Their endeavours have brought about the distinguishing proof of a novel nitrobenzothiazoles (cBT) (Fig. 7) that are non-mutagenic, demonstrate an enhanced wellbeing profile as observed in mammalian cytotoxicity and CYP restraint thinks about, and have amazing antimycobacterial properties [58]. Authors trust that this work will change the way nitroarenes are seen amid the lead-era process [59]. They visualize that, their discoveries will affect the revelation and effective clinical advancement of compounds for the treatment of neglected diseases, for example, leishmaniasis and Chagas disease, for which a significant number of promising nitroarenes have been accounted for as lead [60].

## Structural studies of DprE1 in complex with noncovalent inhibitors

Basic characterization of TCA1 in complex with *M. tuberculosis* DprE1 uncovered that TCA1 is situated in the focal cavity of DprE1 in a boomerang-like compliance as on account of the covalent inhibitors, with the thiophene moiety arranged somewhere down in the hydro-phobic pocket at the base of the dynamic site [5]. Likewise with the covalent inhibitors, this cooperation has all the earmarks of being vital for the official. Besides, TCA1 is kept up by polar contacts between the carboxamido group and thiazole nitrogen of TCA1 and K418 of *M. tuberculosis* DprE1 [40]. Additionally, the carbamate moiety forms van der Waals interaction critical for the stabilisation of the compound with the phenyl ring of Y314 [18].

## TCA1

**Fig. 8:**
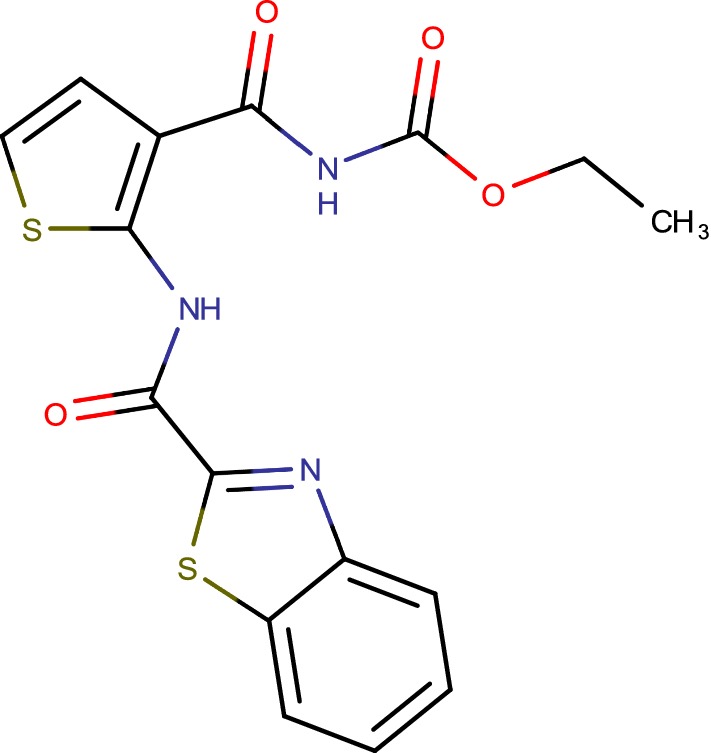
TCA 1

A cell-based phenotypic assay for inhibitors of biofilm development in mycobacteria distinguished the molecule TCA1 (Fig. 8), which has bactericidal activity against both multidrug resistance, extensively drug resistance *Mycobacterium tuberculosis* and affect Mtb in vitro in conjunction with rifampicin or isoniazid. Furthermore, TCA1 has bactericidal activity against non-replicating Mtb in vitro and is useful in strong and continuous Mtb infections in mouse models, both alone and joined with rifampicin or isoniazid [61]. Transcriptional investigation uncovered that TCA1 down-manages qualities known to be associated with Mtb ingenuity. Traditional techniques distinguished decaprenylphosphoryl-β-d-ribofuranose oxidoreductase DprE1, enzyme engaged with cell wall and molybdenum cofactor biosynthesis, separately, as targets in charge of the activity of TCA1 [28]. TCA1 works by a special system including down-direction of determination qualities and restraint of both cell wall and MoCo biosynthesis [42]. Without a hesitation, authors have recognized a compound with great serum half-life that has fantastic exercises under both high-impact and anaerobic conditions (MIC50 esteems are 0.3 and 1.5 μg/mL, individually). Future work will concentrate on extra enhancements in the in vivo movement of this particle and definite unthinking investigations, including endeavours to confine extra safe mutants under changed development conditions [40]. This work underscores the energy of cell-based phenotypic screens to reveal atoms with components of activity that give one of a kind ways to deal with the treatment of human disease condition [27].

## TBA-7371 (1–4 azaindoles)

**Fig. 9:**
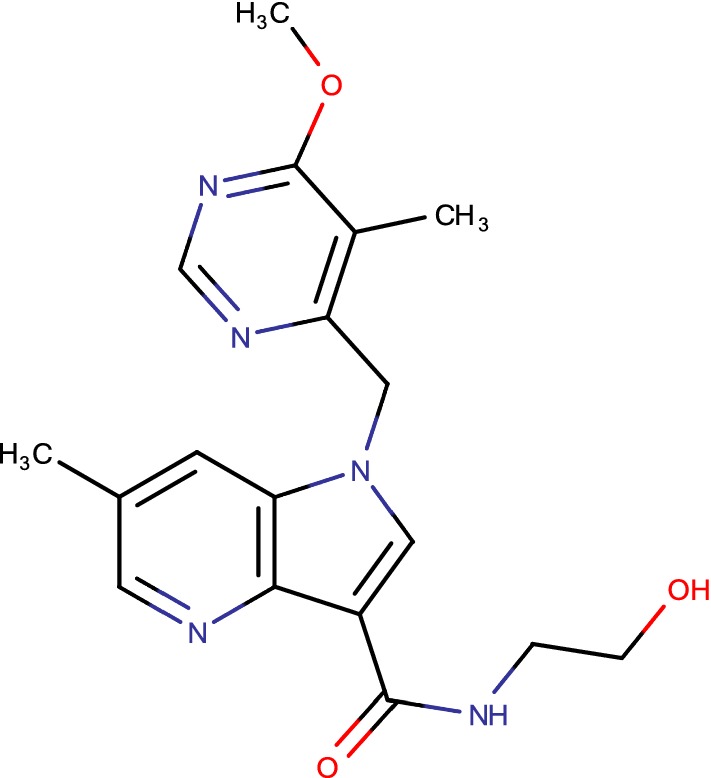
TBA-7371 (1–4 azaindoles)

New effective compounds with novel mechanism of action against multidrug-resistance (MDR) and extensively drug resistance (XDR) *Mycobacterium tuberculosis* are critically required to battle the worldwide tuberculosis (TB). With this, announcement of 1,4-azaindoles (Fig. 9), a promising class of compounds with potent antitubercular activity through noncovalent inhibition of decaprenylphosphoryl-d-ribose 2-epimerase (DprE1) [35]. Further, this series was studied to enhance its physicochemical properties and pharmacokinetics in mice. Here, authors depicted the clinical potential of these series which has strong cell action, capability in mouse, rodent unending TB contamination models and negligible in vitro hazards [40].

## 4-AQs (cmp-3)

**Fig. 10:**
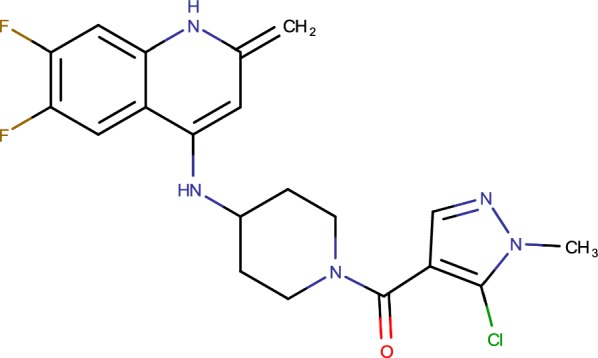
TBA-7371 (1–4 azaindoles)

4-Aminoquinolone piperidine amides (AQs) (Fig. 10) were recognized as a novel platform for antitubercular drug discovery, beginning from an entire cell screen, with intense antibiotic action on *Mycobacterium tuberculosis*. Investigations of the base inhibitory compounds, trailed by entire genome sequencing of mutants raised against AQs, recognized decaprenylphosphoryl-β-d-ribose 2′-epimerase (DprE1) as the essential target for the antitubercular action [51]. AQs have magnificent lead properties and great in vitro pharmacological profile [62]. Despite the fact that, platform began off as an only dynamic compound with direct power from the entire cell screening, structure–activity relationship of the framework prompted mixes with effective DprE1 restraint (IC50 < 10 nM) alongside strong cell action (MIC = 60 nM) against Mtb [36].

## PyrBTZ02 (8-pyrrole-BTZ)

**Fig. 11:**
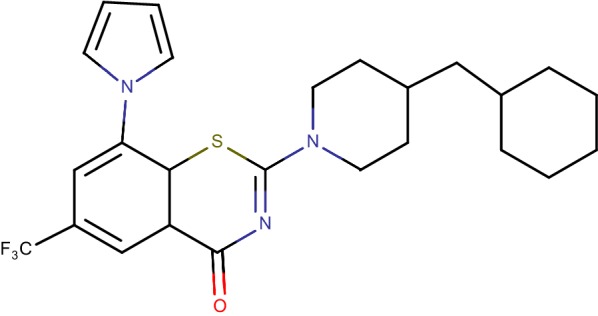
PyrBTZ02 (8-pyrrole-BTZ)

8-Nitro-benzothiazinones (Fig. 11) has shown nano molar in vitro bactericidal action against *Mycobacterium tuberculosis*. Structure–activity relationship (SAR) think about uncovered the 8-nitro group of the BTZ platform to be essential for the system of activity, which includes development of a semimercaptal bond with Cys387 in the dynamic site of DprE1 [29]. To date, substitution of the 8-nitro assemble has prompted loss of antimycobacterial action. Here reported that, union and portrayal of the pyrrole-benzothiazinones PyrBTZ01 and PyrBTZ02, non-nitro-benzothiazinones that hold critical antimycobacterial action, these derivatives repress DprE1 with half inhibitory focus. The most encouraging compound, PyrBTZ01, did not indicate adequacy in a mouse model of extreme tuberculosis, proposing that BTZ-interceded defeating through DprE1 restraint requires covalent bond development [27]. The pyrrole-benzothiazinone analogues represented here (specifically, PyrBTZ01 and PyrBTZ02) gives new bits of knowledge into the substance and pharmacological requirements for DprE1 restraint in mycobacteria. These mixes are the primary non-nitro-benzothiazinones that show critical mycobacterial action in vitro [37].

## 1,2,4-Triazole containing 1,4-BTZ derivatives (cmp-6c)

**Fig. 12:**
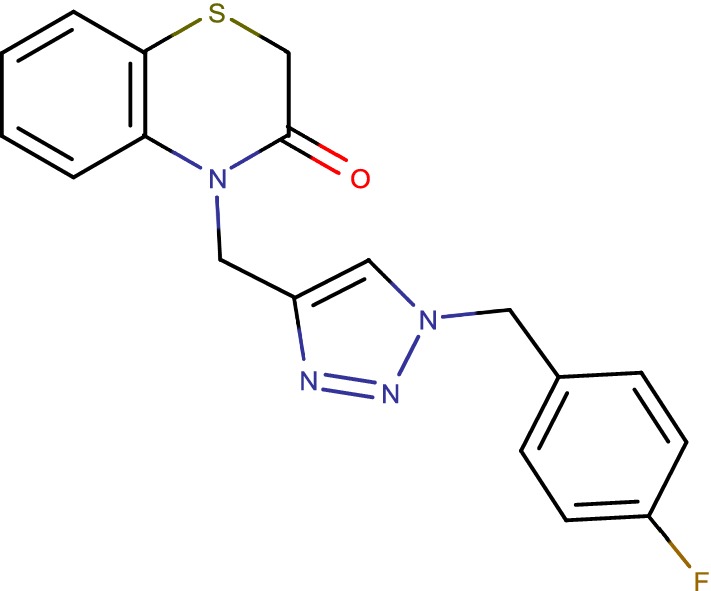
1,2,4-Triazole containing 1,4-BTZ derivatives (cmp-6c)

Looking for new active molecules against *Mycobacterium tuberculosis* H37Ra and *M. bovis* BCG, a library of benzothiazinone based 1,2,3-triazoles (Fig. 12) has been effectively prepared by means of snap science approach [42]. The consequences of the in vitro and in silico, think about propose that the triazole joined benzothiazinone may have the perfect auxiliary pre requisites for improvement of novel compounds [41].

## 1,3-BTZ azide

**Fig. 13:**
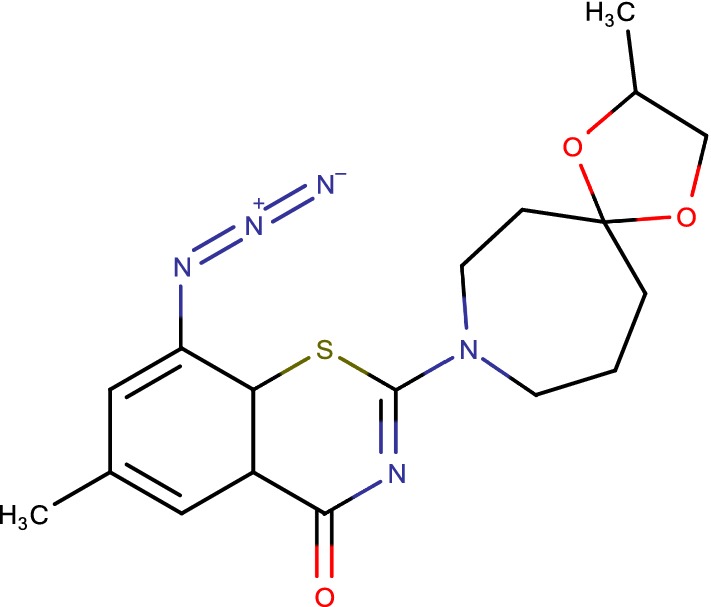
1,3-BTZ azide

Electron lacking nitroaromatic derivatives, for example, BTZ043 and PBTZ169, and related analogues, are promising new class of antitubercular. Thus authors reported the plan of 1,3-benzothiazinone azide (BTZ-N3) (Fig. 13) and related snap science items in light of the atomic method of enactment of BTZ043 [26]. Authors computational docking concluded that BTZ-N3 ties in the basically same pocket as that of BTZ043. Consequent enzymatic investigations with recombinant DprE1 from Mtb took after by MIC assurance in NTB1 strain of Mtb unequivocally demonstrated that BTZ-N3 is a successful reversible and noncovalent inhibitor of DprE1 [39].

## Benzothiazolylpyrimidine-5-carboxamides (cmp-7a)

**Fig. 14:**
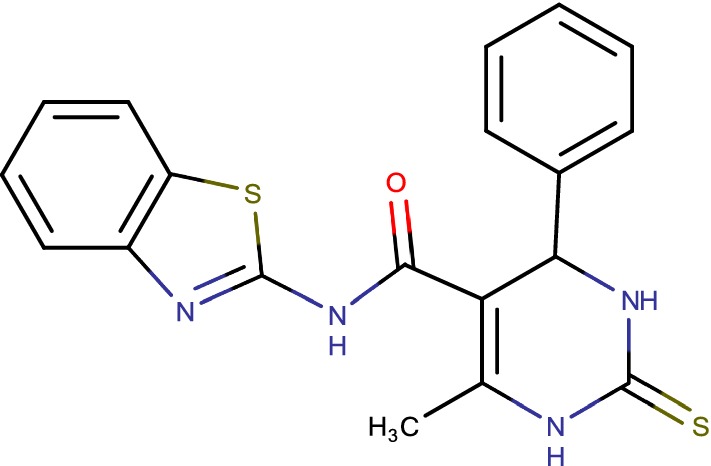
Benzothiazolylpyrimidine-5-carboxamides (cmp-7a)

Decaprenylphosphoryl-b-d-ribose 20-epimerase (DprE1) is a potential target for advancement of antitubercular agents. Structure based drug discovery approach yielded twenty novel analogues of benzothiazolylpyrimidine-5-carboxamides (Fig. 14) which were rewritten by one pot reaction including benzothiazolyl oxobutanamide, thiourea and substituted fragrant benzaldehydes [27].

## Pyrazolopyridones (cmp-19)

**Fig. 15:**
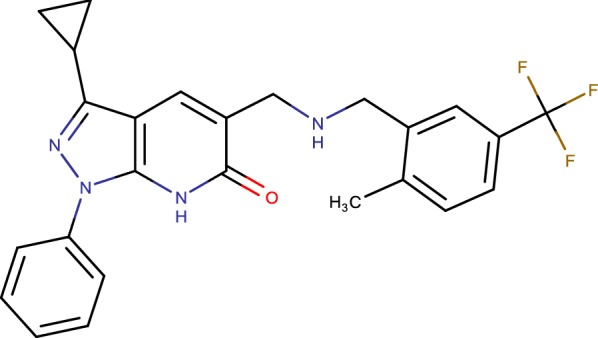
Pyrazolopyridones (cmp-19)

A novel pyrazolopyridone (Fig. 15) class of inhibitors was recognized from entire cell screening against *Mycobacterium tuberculosis* (Mtb) [33]. The arrangement shows surprizing antibacterial activity in vitro. The huge balance of minimum inhibitory concentration (MIC) against Mtb strain overexpressing the Rv3790 quality proposed the objective of pyrazolopyridones to be decaprenylphosphoryl-β-d-ribose-2-epimerase (DprE1) [24]. Docking studies at the dynamic site propose that the arrangement can be additionally differentiated to enhance the physicochemical properties without bartering the antimycobacterial activity [55]. The pyrazolopyridone class of inhibitors offers an appealing non-nitro lead compounds focusing on the fundamental DprE1 inhibitor for the disclosure of novel antimycobacterial specialists to treat resistant strains of Mtb [59].

## Conclusion and future perspectives

Tuberculosis is one of the potential threat to entire mankind as per the history. The numbers of WHO supports global burden of this infection which is increasing drastically. Among the millions of unexplored targets for antitubercular drug discovery, DprE1 is recently came out. Specifically, Nitro group of synthesized compounds gets reduced to nitroso and then it forms adduct with Cys387 residue to exhibit DprE1 inhibitory activity. Very few amount of protein was isolated and studied for specific inhibitory activity. There are few derivatives which were reported in past decade with potential DprE1 inhibitory activity. Even though they have shown DprE1 inhibition but none of them has passed phase II trials. Those inhibitors were covalent as well as non-covalent too. The situation is alarming, so there is strict need to explore this target by designing novel potential analogues to combat drug resistance *Mycobacterium tuberculosis* at the earliest to serve the humanity. There is a strong possibility that DprE1 inhibitors might be active against DprE2 because of the crystal structure of enzyme. In future, researchers have wide scope to work on that with two approaches viz., by designing potent DprE1 inhibitors which may acts against DprE2, by comparing mutants in DprE2.
